# Stable expression of constitutively-activated STAT3 in benign prostatic epithelial cells changes their phenotype to that resembling malignant cells

**DOI:** 10.1186/1476-4598-4-2

**Published:** 2005-01-12

**Authors:** Hosea F Huang, Thomas F Murphy, Ping Shu, Arnold B Barton, Beverly E Barton

**Affiliations:** 1 Division of Urology, Department of Surgery, UMDNJ-New Jersey Medical School,185 S. Orange, Ave., Newark NJ, 07103 USA; 2 Department of Microbiology & Molecular Genetics, UMDNJ-New Jersey Medical School, 185 S. Orange, Ave., Newark NJ, 07103 USA

## Abstract

**Background:**

Signal transducers and activators of transcription (STATs) are involved in growth regulation of cells. They are usually activated by phosphorylation at specific tyrosine residues. In neoplastic cells, constitutive activation of STATs accompanies growth dysregulation and resistance to apoptosis through changes in gene expression, such as enhanced anti-apoptotic gene expression or reduced pro-apoptotic gene expression. Activated STAT3 is thought to play an important role in prostate cancer (PCA) progression. Because we are interested in how persistently-activated STAT3 changes the cellular phenotype to a malignant one in prostate cancer, we used expression vectors containing a gene for constitutively-activated STAT3, called S3c, into NRP-152 rat and BPH-1 human benign prostatic epithelial cells.

**Results:**

We observed that prostatic cell lines stably expressing S3c required STAT3 expression for survival, because they became sensitive to antisense oligonucleotide for STAT3. However, S3c-transfected cells were not sensitive to the effects of JAK inhibitors, meaning that STAT3 was constitutively-activated in these transfected cell lines. NRP-152 prostatic epithelial cells lost the requirement for exogenous growth factors. Furthermore, we observed that NRP-152 expressing S3c had enhanced mRNA levels of retinoic acid receptor (RAR)-α, reduced mRNA levels of RAR-β and -γ, while BPH-1 cells transfected with S3c became insensitive to the effects of androgen, and also to the effects of a testosterone antagonist. Both S3c-transfected cell lines grew in soft agar after stable transfection with S3c, however neither S3c-transfected cell line was tumorigenic in severe-combined immunodeficient mice.

**Conclusions:**

We conclude, based on our findings, that persistently-activated STAT3 is an important molecular marker of prostate cancer, which develops in formerly benign prostate cells and changes their phenotype to one more closely resembling transformed prostate cells. That the S3c-transfected cell lines require the continued expression of S3c demonstrates that a significant phenotypic change occurred in the cells. These conclusions are based on our data with respect to loss of growth factor requirement, loss of androgen response, gain of growth in soft agar, and changes in RAR subunit expression, all of which are consistent with a malignant phenotype in prostate cancer. However, an additional genetic change may be required for S3c-transfected prostate cells to become tumorigenic.

## Introduction

**Figure 1:**
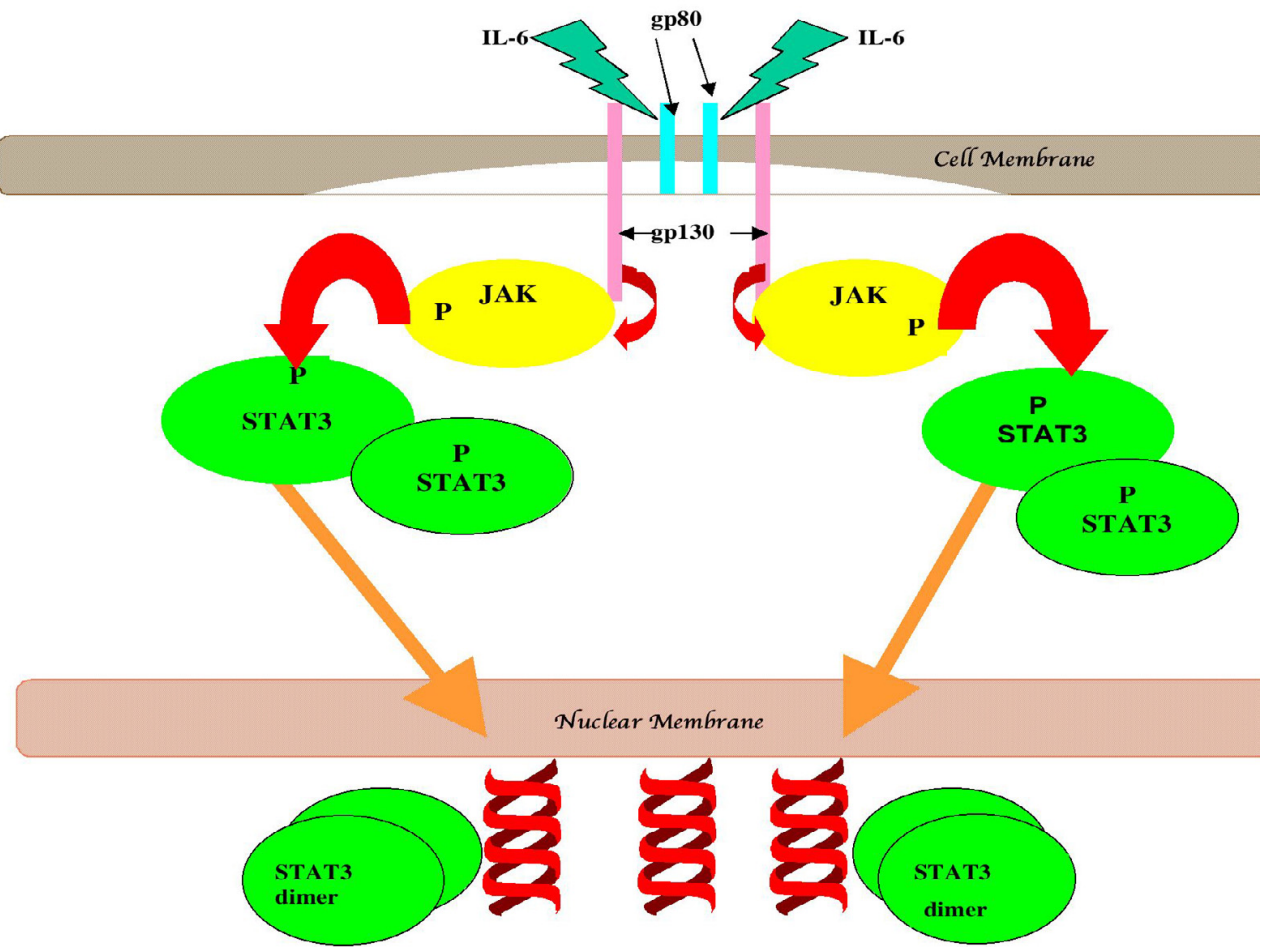
An example of cytokine-mediated activation of STAT3. In this example, IL-6-induced binding to its receptor leads to homodimerization of the receptor, which in turn leads to autophosphorylation of the cytosolic domain of gp130; this in turn causes the phosphorylation of one of 3 kinases, JAK1, JAK2, or Tyk 2. The activated up-stream kinase phosphorylates STAT3, which allows for dimerization of STAT3; only the dimer can translocate and dock to DNA at target genes, thereby directing transcription.

Signal transducers and activators of gene transcription (STATs) are, as their name suggests, proteins that regulate gene expression by affecting transcription. They are part of the signal transduction pathway used by many growth factors and cytokines, and are activated by phosphorylation of tyrosine and serine residues by up-stream kinases [1]. For example, signaling by IL-6 and other members of this cytokine family generally induces phosphorylation of STAT3 [1,2]. In the example given in Figure 1, IL-6-induced binding to its receptor leads to homodimerization of the receptor, which in turn leads to autophosphorylation of the cytosolic domain of gp130; this in turn causes the phosphorylation of one of 3 kinases, JAK1, JAK2, or Tyk 2. The activated up-stream kinase phosphorylates STAT3, which allows for dimerization of STAT3 although this concept is currently being revisited, since it has been shown in hepatic cells under inflammatory stress, there is evidence for STAT3 association on lipid rafts prior to phosphorylation [3,4] in association with chaperone proteins such as Hsp90 (reviewed in [5]); however only the dimer form of STAT3 can translocate and bind to DNA at specific binding sites, thereby directing transcription of target genes. In benign cells, the signaling by STAT3 is under tight regulation, so that the signal delivered to the cell is transient. However aberrant signaling by STAT3 has been noted in many types of malignancies, such as myeloma, head and neck cancer, breast cancer, and prostate cancer [6-9]. Such persistent signaling by IL-6 leading to aberrant activation of STAT3 is thought to play a role in neoplastic progression of prostate cells [10]. Importantly, we and others have shown that malignant prostate cells expressing persistently-activated STAT3 become dependent upon this transcription factor for survival, resulting in apoptosis [11-13]. Thus, persistently-activated STAT3 fulfills the criteria of a proto-oncogene [14,15].

Prostate cancer (PCA) is the second most frequently diagnosed non-cutaneous malignancy in American males, affecting approximately 35% of them according to recent data [16,17]. This translates into approximately 35,000 deaths last year in the United States alone; 189,000 new cases were diagnosed in 2002 and over 220,000 cases were projected for 2003 [18,19]. Moreover, in a recent report the authors claimed that 30% of male mortality overall may be due to prostate cancer [20]. For the most effective therapy with the fewest side-effects, a thorough understanding of the genes involved in the neoplastic process is essential. Androgens are known to play a critical role in the tumorigenic process, with activity mediated by the androgen receptor. Initially, prostate cancers are androgen-sensitive (that is, they cease growing when deprived of androgens or when treated with androgen receptor antagonists, such as flutamide or bicalutamide), and therefore most patients respond to androgen ablation therapy. However, there are side-effects to this therapy that make it unpleasant for the patient [21]. Even with androgen ablation therapy, the disease often recurs and when it does, it usually becomes androgen-insensitive or hormone-refractory [22]. There is evidence that STAT3 activation via IL-6 plays a role in the conversion of normal prostate cells to prostate cancer cells, and from androgen-responsive to the androgen insensitive phenotype [10,23,24]. The progression to androgen-independence has been found to be associated with IL-6, with c-myc expression, and with insulin-like growth factors, all of which can signal through the activation of STAT3 [25-28]. STAT3 is negatively regulated by a retinoid-sensitive protein, GRIM-19, which may explain the positive effects retinoids show against prostate cancer cells in vitro [29-31]. Retinoid therapy for the treatment of prostate cancer is currently being tested, due to the ability of these compounds to rapidly induce apoptosis [32]. Indeed, the recent addition of Taxotere to the pharmacopeia for prostate cancer may well be due to its demonstrated effect on retinoid receptors [33]. The regulation of the expression of the 3 retinoid receptors type A (RAR-α, -β, and -γ) in the progession to prostate cancer has been partially addressed by Richter, et al., who showed the differential effects of all-*trans *retinoic acid in human prostate cancer lines [34,35]

To this end we are studying the oncogenic role of STAT3 activation in rat prostate epithelial cell lines NRP-152 [36] and human benign prostatic hyperplasia line BPH-1 [37,38]. Our main hypothesis is that constitutively-activated STAT3 (cSTAT3) plays an essential role in the development of PCA and the maintenance of the malignant phenotype. Because prostate epithelial cells become hypertrophic, but rarely malignant, they are useful for studying the progression to neoplasia to see how a relatively transformation-resistant cell type becomes neoplastic through cSTAT3. We previously determined that STAT3 was constitutively phosphorylated (hence activated) in malignant NRP-154 but not in NRP-152 cells, even when the NRP-152 cells were treated with testosterone [10]. We hypothesized that cSTAT3 may account for the tumorigenicity of NRP-154 cells, and therefore may play a determining role in the progression from hyperplasia to neoplasia. To test our hypothesis, we transfected a plasmid containing a mutated gene for STAT3 known as S3c, in which a Cys residue was substituted for an Ala residue, thereby allowing the dimerization of the mutated STAT3, which can then translocate across the nuclear membrane and effect gene transcription in much the same way as the phosphorphylated wild-type STAT3 gene product [14,39] into NRP-152 and BPH-1 cells. We then examined the phenotype of the selected transfected cells after cloning by limit dilution. Our results, indicating that NRP-152 and BPH-1 cells underwent changes in phenotype consistent with that of malignant cells, are presented here.

## Results

### Selection of Transfected NRP-152 and BPH-1 Cells

Two weeks after transfection with either pIRES or pIRES-S3c and selection with G418, no surviving cells were observed in the wells that received Clonfectin only. Growth of cells was observed in all wells that received either of the plasmids plus Clonfectin. Transfected cells were expanded for further analysis in complete medium. A summary of cells and clones and what their phenotypes were is given in Table 1. To summarize briefly, since the full results will be discussed in this section, we observed the following changes:

**Table 1:** Summary of transfected cells

		**Growth Factor**	**G418**	**FLAG**	**EGFP**	**Growth In**
		
**Cell**	**Plasmid**	**Dependence**	**Sensitivity**	**Epitope**	**Expression**	**Simple Medium**
NRP-152	none	x	x	-	-	-
152-pIRES	pIRES-EGFP	x	-	-	x	-
152-S3c	pIRES-S3c	-	-	x	x	x
						
BPH-1	none	n/a	x	-	-	n/a
BPH-pIRES	pIRES-EGFP	n/a	-	-	x	n/a
BPH-S3c	pIRES-S3c	n/a	-	x	x	n/a

The table summarizes the cells used or made in the experiments described. Cells were transfected with the indicated plasmid, as described in Materials & Methods. G418 was added at 400 μg/ml. Growth factors were those added to 152 medium, but not to 154 medium, as described in Materials and Methods, for NRP-152 cells and transfectants.

X = presence of characteristic; --- = absence of characteristic.

NRP-152 cells require a variety of growth factors and additives in their medium (see Materials and Methods Section; [36]); 152-pIRES cells (NRP-152 cells transfected with pIRES-EGFP) required the same medium as NRP-152 cells. But 152-S3c cells grew in DMEM/Ham's F12 supplemented only with 10% newborn calf serum. Moreover, 152-S3c cells expressed EGFP (as did 152-pIRES, which was expected since they were transfected with pIRES-EGFP) and the FLAG epitope, which is part of the S3c gene [40]. Both 152-pIRES and 152-S3c cells grew in the presence of G418.

BPH-1 cells grow in RPMI-1640 supplemented with bovine serum; therefore this line does not have growth factor dependence to begin with. BPH-pIRES and BPH-S3c cells, aside from exhibiting G418 resistance, expressed EGFP, but only BPH-S3c expressed the FLAG epitope of the S3c gene. The evidence for these observations given in Table 1 is presented in the rest of this section.

### Expression of FLAG and EGFP in 152-S3c and BPH-S3c Cells Was Observed After Transfection and Selection with Antibiotics

**Figure 2:**
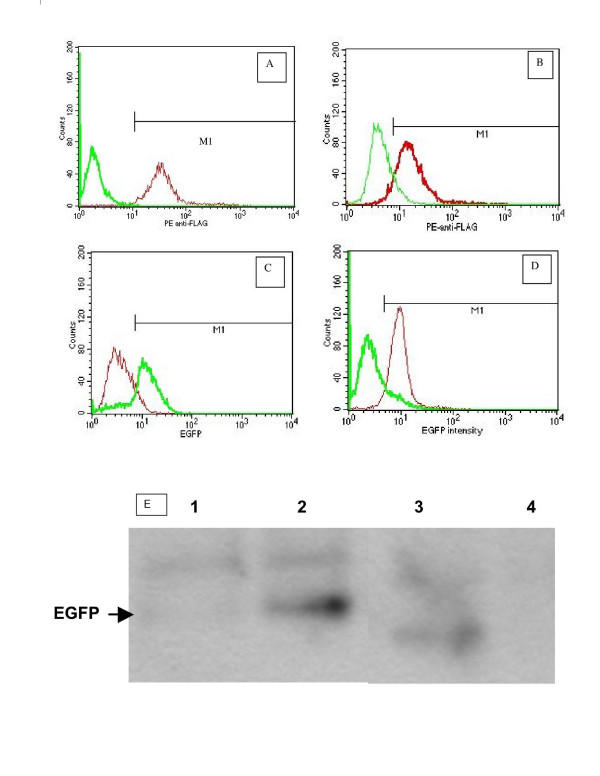
FLAG and EGFP expression in representative NRP-152 and BPH-1 clones transfected with either pBABE-S3c or pIRES-S3c. NRP-152 and BPH-1 cells were transfected with pBABE-S3c or pIRES-S3c, which bear the FLAG epitope on the S3c gene. Clones were derived by limit dilution, as described in Materials & Methods. **Panels A–D: **In all histograms, the marker M1 sets the region of positively fluorescent cells for determining the percent positive cells. **Panels A & B: **Fixed cells were permeabilized and stained with anti-FLAG M1 Ab (Sigma), as described in Materials & Methods. Controls for staining were included, as described. **Panel A: **Transfected NRP-152 cells. Thin line = 152-S3c; thick line = NRP-152. **Panel B: **Transfected BPH-1 cells. Thin line = BPH-1; thick line = BPH-S3c. **Panels C & D**: NRP-152 and BPH-1 cells transfected with pIRES-S3c were analyzed for EGFP fluorescence, following selection. **Panel C: **Transfected NRP-152 cells. Thin line = NRP-152; thin line = 152-S3c. **Panel D**: Transfected BPH-1 cells. Thick line = BPH-1; thin line = BPH-S3c. **Panel E: **Immunoprecipitation followed by Western blot showing EGFP expression in transfected NRP-152 and BPH cells. Note the lack of EGFP bands for parental lines NRP-152 and BPH-1, whereas EGFP was detected using EGFP-specific Ab (Pharmingen) in lanes for 152-S3c and BPH-S3c. **Methods: **NRP-152, 152-S3c, BPH-1, and BPH-S3c cells were lysed in buffer. Equal amounts of protein in cell lysates were pre-cleared with Protein A/G beads, then precipitated with anti-FLAG AB plus Protein A/G beads with rotation in the cold. The pelleted beads plus proteins were separated on 12% SDS gels, transferred to PVDF membranes, then blotted with Ab to EGFP. Enhanced chemifluorescence was used to reveal the 27 kD bands corresponding to EGFP.

After no viable cells were observed following antibiotic treatment, we analyzed transfected cells for the presence of the markers flanking the S3c gene on the plasmids used, FLAG and EGFP. The analyses were done by flow cytometry on a FACScan; also by Western blot using specific Abs, and the results are presented in Figure 2. In Panels A through D, the mean fluorescence intensities of representative clones of 152-S3c and BPH-S3c cells stained with monoclonal Ab to FLAG plus fluorescent goat anti-mouse F(ab_2_)', as well as the enhanced green fluorescent protein fluorescence intensities of transfected cells, are shown. **Panel A **displays the anti-FLAG fluorescence intensity of 1 representative clone of 152-S3c (thin line) compared to untransfected NRP-152 cells (thick line); approximately 95% of the 152-S3c cells stained with the anti-FLAG antibody. Similary, **Panel B **shows the fluorescence intensity of anti-FLAG-stained BPH-1 cells (thin line) compared to anti-FLAG-stained BPH-S3c clone (thick line), where approximately 76% of the BPH-S3c cells stained with the anti-FLAG antibody. **Panels C and D **display the EGFP fluorescence for clones of 152-S3c and BPH-S3c cells, compared with untransfected cells, respectively. In **Panel C**, the thick line shows the fluorescence intensity of EGFP in 152-S3c and the thin line shows the lack of EGFP fluorescence in the untransfected NRP-152 cells. Approximately 67% of the 152-S3c cells showed EGFP fluorescence. In **Panel D**, the thin line shows the EGFP fluorescence intensity of BPH-S3c cells, while the thick line shows it for untransfected BPH-1 cells. Approximately 45% of the BPH-S3c cells showed fluorescence due to EGFP. We concluded that in addition to antibiotic resistance, the transfected cells expressed markers flanking the S3c gene, and therefore we could attribute any change in phenotype of the cells to the expression of the S3c, in comparison to the vector-transfected cells. **Panel E **shows the results of immunoprecipitation with anti-FLAG Ab, followed by Western blot to detect EGFP. We used anti-FLAG Ab for the immunoprecipitation because (1) a S3c-specific Ab is not available, and (2) because all cells express STAT3. Thus, because expression of FLAG equates with expression of S3c specifically, immunoprecipitating with anti-FLAG would reveal the S3c-expressing cells. As seen in Figure 2E, the bands corresponding to 27 kD EGFP are visible only in the lanes from 152-S3c and BPH-S3c cells, while no EGFP bands are visible in the bands from the parental lines NRP-152 and BPH-1 cells. Since the EGFP gene is 3' to the S3c gene in the pIRES-S3c plasmid we constructed (the plasmid codes for a bicistronic message with 1 promoter for EGFP and S3c), these results confirm the flow cytometry data shown in Panels A through D.

### 152-S3c Cells Grew in the Absence of Exogenous Growth Factors

**Figure 3:**
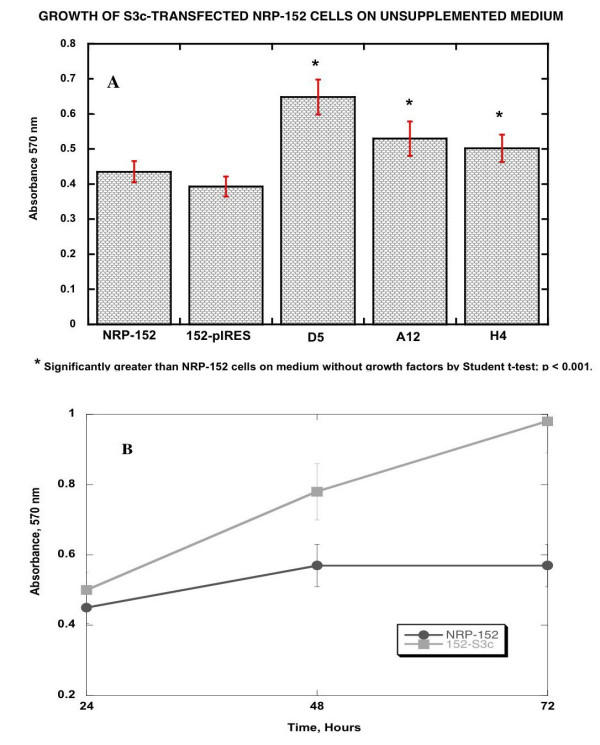
Growth of NRP-152, NRP-154, 152pBABE, and 152-S3c clones on 154 medium compared to growth on 152 medium. 10^3 ^cells were seeded in microtiter wells, in the indicated medium. After incubation for 48 hr, MTT (15 μl at 25 μg/ml) were added to each well, and incubation was continued for 4 hr more. The formazan was dissolved in 0.1% SDS, and the absorbance was quantified on a DynaTech plate reader at 570 nm. Unpaired Student t-tests (InStat3 software) were performed to assess the statistical significance of the growth of S3c-transfected cells relative to pBABE transfected and untransfected NRP-152 cells. **Panel A: **Comparison of growth as measured by MTT absorbance at 48 hours; **Panel B: **Comparison of growth rates over 72 hours.

To demonstrate that 152-S3c cells grew in the absence of growth factors required by untransfected NRP-152 cells, transfected and untransfected NRP-152 cells were grown in microtiter wells. Proliferation was quantified by the oxidation of MTT after 48 hr. Figure 3 shows the results of these experiments. NRP-152 and 152-pIRES cells grew more slowly in unsupplemented 154 medium than they did in 152 medium. However, 152-S3c cells (3 representative clones, D5, A12, and H4, are shown) grew nearly as well in 154 medium as in 152 medium, and grew significantly better in 154 medium than either NRP-152 or 152-pBABE cells (p < 0.001; Figure 3A). Therefore, clones of 152-S3c cells, stably transfected with pBABE-S3c, grew in vitro as if they lost the requirement for additional growth factors in the cell culture medium.

### Stable Expression of S3c in BPH-1 Cells Resulted in STAT3-Dependence for Survival

**Figure 4:**
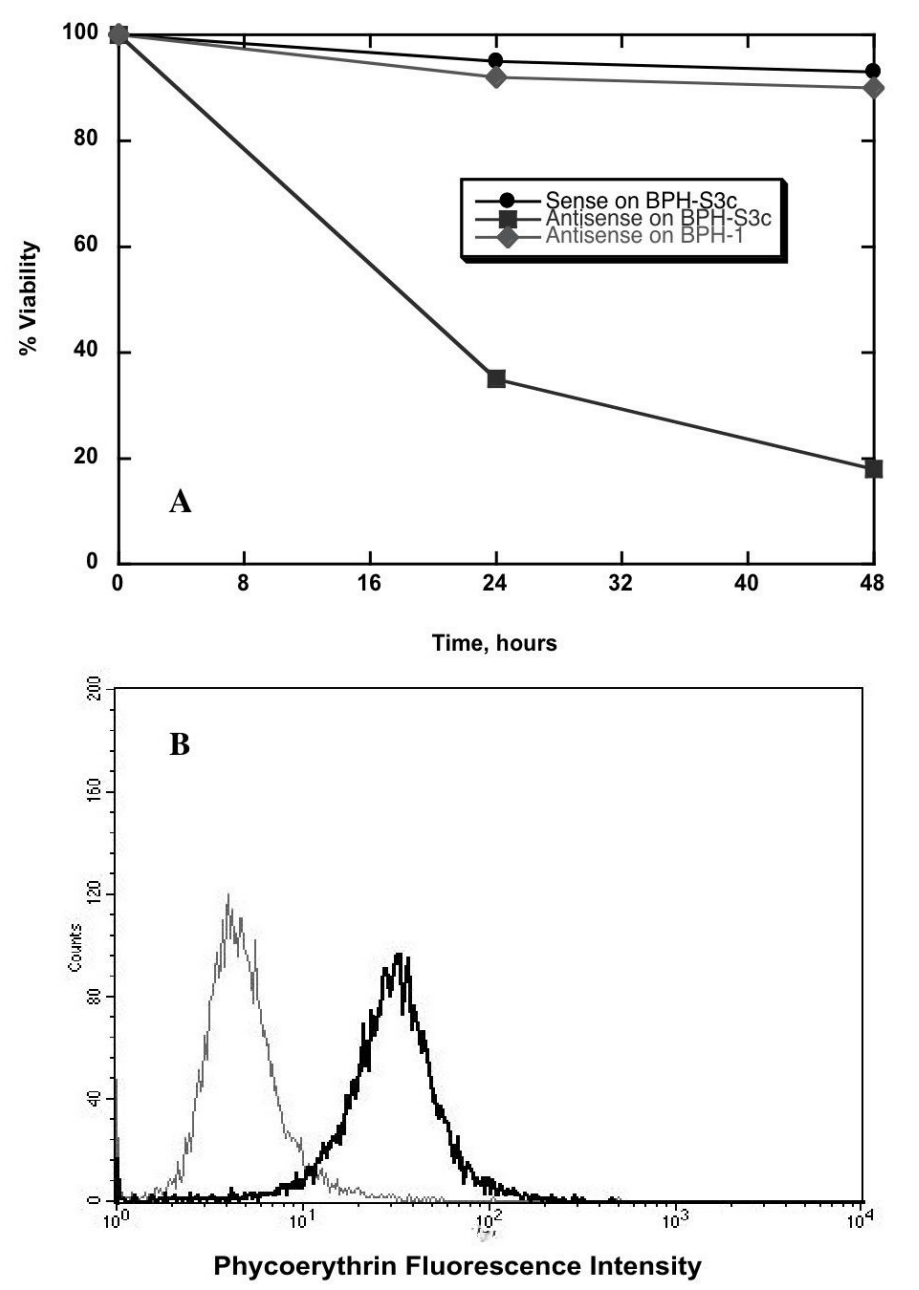
Functional activity of STAT3 in S3c-transfected cells. **Panel A: **To show the functional activity of STAT3 expressed by the S3c gene, BPH-1 cells stably transfected with pIRES-S3c were treated with either 125 nM sense or antisense STAT3 oligonucleotide. Percent viability over time was determined by staining with propidium iodide, then quantifying fluorescence on a FACScan flow cytometer. **Panel B: **Treatment with 125 nM antisense STAT3 oligo reduced the amount of intracellular STAT3 protein in the clone of BPH-S3c cells shown in 3A. Twenty-four hours after transfection, BPH-S3c cells were harvested, fixed, and permeabilized, then stained with antibody to STAT3, as described in Materials and Methods. Quantification was performed on a FACScan flow cytometer. The black line indicates the amount of intracellular STAT3 in BPH-S3c cells treated with sense STAT3, while the grey line shows the amount of STAT3 in BPH-S3c cells given antisense STAT3. STAT3 expression was reduced by 66% in this experiment.

In order to show that the persistent expression of activated STAT3 was required for the survival of the transfected cells, as we have previously shown for hormone-refractory prostate cancer cells lines [11,12], we transfected pIRES-S3c into human BPH-1 cells [38] for studies with antisense STAT3 oligonucleotides. We used BPH-1 cells and transfected lines only for these experiments, because the antisense oligonucleotide was designed for use in human cells, and we wanted to maximize the efficacy of the antisense oligonucleotide. Figure 4 shows that transfection of 125 nM of sense STAT3 oligonucleotide decreased viability by only 5% at 48 hours, whereas transfection of the same amount of antisense STAT3 oligonucleotide decreased viability to 18% at 48 hours. Furthermore, transfection of antisense STAT3 oligonucleotide into untransfected BPH-1 cells did not decrease viability any more than did transfection of sense oligonucleotide. Figure 4B shows that 24 hours after transfection with 125 nM of antisense STAT3, BPH-S3c cells displayed a 66% reduction in intracellular STAT3 protein levels. We concluded from these experiments that the S3c expressed in BPH-S3c cells was functionally active, and that BPH-S3c cells were dependent upon continued STAT3 expression for their very survival, just like hormone-refractory prostate cancer cell lines [11,13]. These data are more evidence for a profound difference in phenotype between BPH-1 cells and BPH-S3c cells.

### 152-cS3 Cells Have Decreased Expression of RAR-β and -γ mRNA, and Increased Expression of RAR-α mRNA

**Figure 5:**
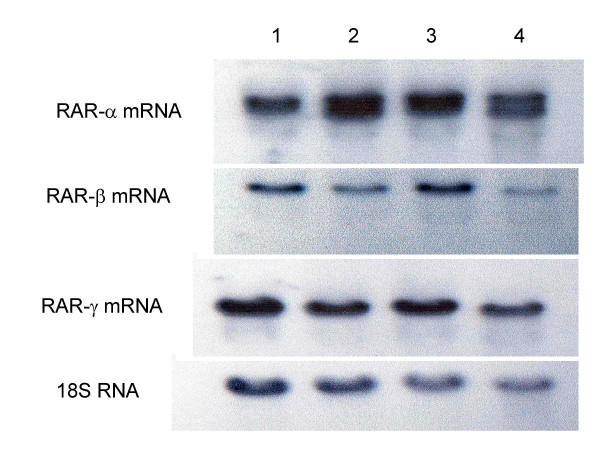
S3c expression inhibited RAR-β and -γ expression and increased expression of RAR-α in NRP-152 cells. **Panel A: **Effect of S3c on RAR-β mRNA levels. **Panel B: **Effect of S3c on RAR-γ mRNA levels. **Panel C: **Effect of S3c on RAR-α mRNA levels. NRP-152, NRP-154, NRP-pBABE, and 152-S3c cells were grown to confluence, and RNA was harvested as described in Materials & Methods. Electrophoretic separation of RNA was followed by transfer to nitrocellulose, then hybridization with ^32^P-labeled probe, followed by autoradiography. Lane 1 = NRP-152 (rat benign prostatic hyperplasia line); lane 2 = NRP-154 (rat prostatic carcinoma line); lane 3 = 152-pBABE; lane 4 = 152-S3c. The comparison to 18S RNA is shown for each.

In prostate cancer cell lines and archived specimens, we previously found that RAR-β and -γ have decreased mRNA levels, while RAR-α mRNA increased, relative to non-malignant prostate cell lines and the normal margins of the same specimens [34,35]. This finding is also true of NRP-152 and NRP-154 cells: the expression of RAR-β and -γ is decreased in NRP-154 cells relative to NRP-152 cells. In order to see if the same change in retinoic acid receptor subunit expression occurred when S3c is expressed, which is consistent with the malignant phenotype, we did the following experiments. For these, we used 152-S3c and 152-pIRES cells, so that we could compare the RAR levels with those of NRP-154 and parental NRP-152 cells, because these 2 related cell lines are believed to represent two stages in the progression and development of prostate cancer [36,41]. Figure 5 depicts the northern blot hybridization results for RAR-β (Figure 5A) and -γ (Figure 5B) in transfected and untransfected cells. Lane 1 in both panels shows the hybridized mRNA for untransfected NRP-152 cells, while both lanes 2 show the hybridized band for NRP-154 cells. Note the decreased amount of RAR-β and -γ in lanes 2 (from NRP-154 cells, the prostatic carcinoma line) relative to the amount in lanes 1, obtained from NRP-152 cells, the benign prostatic hyperplasia line. Lanes 3 show the hybridized mRNA obtained from NRP-152 cells transfected with the vector, pIRES-EGFP, while the bands displayed in both lanes 4 shows that when NRP-152 cells were transfected with pIRES-S3c, the hybridization of RAR-β and -γ decreased similarly to what is observed in lanes 1 and 2. Figure 5C compares RAR-α mRNA expression in the 4 cell lines: lane 1 again is NRP-152 and lane 2 is NRP-154; there is more mRNA hybridized in lane 2 than in lane 1, and the band appears as a doublet in lane 2 as well. Lane 3 shows the results from NRP-152 cells transfected with pIRES-EGFP, while lane 4 shows the results from NRP-152 transfected with pIRES-S3c: note the similar pattern to that of lanes 1 and 2 – lane 4 shows more hybridization and a doublet band for RAR-α as well. We concluded from these results that transfection of NRP-152 cells with pIRES-S3c, but not pIRES-EGFP, induced a change in RAR mRNA expression that is often observed in prostate cancer cell lines and archived specimens.

### BPH-S3c Cells Were Androgen-Insensitive

In many human prostate cancers, overexpression of the androgen receptor has been noted [42,43]. Therefore, the development of the hormone-refractory state apparently occurs even when there is no disruption of the expression of the androgen receptor, at least in some prostate cells. To clarify these contradictory data and to check for the development of functional androgen-insensitivity, we examined the growth rate of human BPH-1 and BPH-S3c cells in the presence and absence of dihydrotestosterone (DHT), and also DHT in the presence of the antagonist flutamide (F). Our results, presented in Table 2, show that while BPH-1 cells respond to DHT and are blocked by F, the same is not true of BPH-S3c. Thus, the persistent expression of S3c in BPH-1 cells resulted in a functionally androgen-insensitive state for these cells.

**Table 2:** Androgen-Insensitivity is conferred by S3C expression in BPH-1 cells

**Cell**	**nM DHT**	**%Stimulation**	**μM F + nM DHT**		**%Inhibition**
BPH	10	200	**1**	10	97
BPH-pIRES	10	250	**1**	10	99
BPH-S3c	10	2	**1**	10	-4
DU145	10	3	**1**	10	3

Cells were grown in 96-well plates for 72 hr, in the presence or absence of drugs (DHT = dihydrotestosterone; **F = flutamide, bolded and underlined**) as indicated. The MTT assay was used to assess proliferation. %Enhancement = (absorbance + drug/absorbance - drug) × 100; % Inhibition = 1-(absorbance + drug/absorbance - drug) × 100. The responses of DU145 cells, a human prostate cancer cell line that is androgen-insensitive and resistant to flutamide, is shown for comparison.

### 152-S3c Cells Lost Sensitivity to the JAK2 Inhibitor AG490

In non-malignant cells, the activation of STAT3 is effected by a specific upstream kinase, JAK1 or JAK2 or sometimes Tyk2. Previously we had shown that the constitutive activation of STAT3 in NRP-154 cells rendered those cells insensitive to apoptosis induced by the JAK2 inhibitor AG490 [10]. In order to see if insensitivity to AG490 was conferred on 152-S3c cells, we added AG490 to cells and assessed apoptosis 48 hr later by annexin V binding and PI inclusion. Table 3 shows the data we obtained. Whereas NRP-152 and 152-pIRES cells were 45 ± 10% and 38 ± 5% apoptotic, respectively, 48 hr after treatment with 100 μM AG490, only 6.3 ± 3% of 152-S3c cells and 7.5 ± 4% of the NRP-154 cells were apoptotic after 100 μM AG490 treatment. We conclude from these experiments that S3c expression in NRP-152 cells decreased their sensitivity to AG490, which is consistent with what we observed in malignant NRP-154 cells.

**Table 3:** NRP-152 Cells Transfected with S3c lost sensitivity to JAK2 inhibitor AG490

**Cell**	**S3c?**	**Rx**	**μM**	**% Apoptotic +/- SD**
NRP-154	YES	AG490	0	8 ± 4
			100	7.5 ± 5
5152-S3c	YES	AG490	0	7.5 ± 4
			100	6.3 ± 3
152-pIRES	NO	AG490	0	11 ± 2
			100	38 ± 5*
NRP-152	NO	AG490	0	7.5 ± 4
			100	45 ± 10*

Cells were placed in 60 mm wells for 48 hr with compound at the concentrations indicated. Zero concentration of the compound is the vehicle (DMSO) control. At the end of the incubation period, cells were harvested, washed, and stained with FITC-annexin V, to demonstrate apoptotic cells. Quantification of fluorescence was performed on a Becton-Dickinson FACScan flow cytometer using CellQuest software.

* p < 0.005 by Student t-test, compared to vehicle-treated cells.

### 152-S3c Cells Grew in Soft Agar

As an in vitro indication of tumorigenic potential, soft agar cloning assays were performed as described [44]. S3c-transfected cells were compared to NRP-152 and to pIRES-EGFP-transfected cells in these experiments. We observed that 152-S3c cells grew significantly better (p < 0.0001 by 2-tailed Student t-test) in soft agar than either untransfected NRP-152 or pIRES-transfected NRP-152 cells (Table 4). We conclude from these experiments that 152-S3c cells have the potential to form tumors in vivo, whereas it has previously been established that NRP-152 cells are not tumorigenic [36], and we would not expect 152-pIRES cells to be tumorigenic either.

**Table 4:** NRP-152 Cells transfected with S3c grew in soft Agar

**CELL**	**S3c?**	**#WELLS**	**#COLONIES +/- SEM**
			
NRP-152	NO	12	2.6 ± 0.9
152-pIRES	NO	12	5.8 ± 1.8
152-S3c	YES	12	35 ± 4.5*

Cells were placed in 60 mm wells in soft agar for 10 days. Colonies of more than 1 mm in size were counted were counted using an inverted phase contrast microscope.

* p < 0.001 by Student t-test

### Expression of S3c Did Not Confer Tumorigenicity on Benign NRP-152 Cells

Based on our previous data, especially the soft agar cloning data, we expected that 152-S3c cells would form tumors in SCID mice. However, in 3/3 experiments (in two them, Matrigel was used to enhance tumorigenicity of the cells), an average of 1/5 mice developed tumors; these were 1 mm in diameter or less. We chose to use only transfected NRP-152 cells for these experiments, because in certain in vivo environments, untransfected BPH-1 cells have been observed to form tumors [38]. We conclude that while persistent S3c expression altered the phenotype of 2 different benign prostatic hyperplasia lines in ways consistent with the development of the malignant phenotype, an additional change in gene expression may be required for tumorigenicity in prostate cancer development.

## Discussion

We have demonstrated that NRP-152 and BPH-1 cells transfected with a constitutively-activated form of the STAT3 gene, S3c, gained a phenotype which more closely resembled that of NRP-154 cells. Specifically, the transfected cells expressed resistance to the antibiotic G418, and also expressed the FLAG epitope, as revealed by intracellular flow cytometry following staining with anti-FLAG Ab in Figure 2B–C, while Figure 2A shows the FLAG expression in mock transfected cells. As additional evidence of S3c expression, we looked for EGFP expression in 152-pIRES cells, since the bicistronic message from this vector (pIRES-EGFP) places the S3c gene 3' to the EGFP, so that S3c would have to be translated before EGFP is translated. Figure 2D shows the EGFP expression in the same clone whose FLAG expression is shown in Figure 2C. These results were confirmed by immunoprecipitation/Western blot analysis, which is shown in Figure 2E, whereupon cell lysates were precipitated with Ab to the FLAG peptide on the S3c gene, then blotted with anti-EGFP Ab. Only the transfected and selected 152-S3c and BPH-S3c cells revealed EGFP bands, not the parental lines. After obtaining these results, we characterized the phenotype of the transfected cells.

Parental NRP-152 cells are fastidious in their growth factor requirement, whereas NRP-154 cells and 152-S3c clones grew in medium supplemented only with serum (Figure 3). Therefore, we assessed the change in growth of transfected NRP-152 cells by comparing their growth in unsupplemented medium. We found that clones of 152-S3c cells grew nearly as well as NRP-154 cells in simple medium, whereas NRP-152 and 152-pIRES cells grew poorly in the absence of growth factors included in the medium (Figure 3). The change in growth factor requirement is one often observed for neoplastic cells, and is consistent with the role of STAT3 as a proto-oncogene with the capability of transforming benign cells into malignant cells [15,45]. As for dependence on survival of constitutively-activated STAT3, which has been observed in NIH-3T3 transfected with S3c [40] and in hormone-refractory prostate cancer cell lines [11], BPH-S3c cells treated with 125 nM antisense STAT3 oligonucleotides died over time, going from 100% viable to less than 20% viable 48 hours after transfection (Figure 4A); the reduction in viability could be attributed to the effect of antisense STAT3 on STAT3 protein expression, which was reduced by 66% at 24 hours after transfection (Figure 4B). These data mean that like hormone-refractory prostate cancer cells, BPH-1 cells transfected with S3c became dependent upon the continued expression of S3c for their survival.

As for RAR expression, we observed decreased mRNA levels of RAR-β and -γ, but increased RAR-α expression in S3c-transfected NRP-152 cells; the results shown in Figure 5 are consistent with the expression levels of these receptor mRNAs previously observed by us in prostate cancer lines [34] and in prostate cancer patient specimens [35]. These findings are echoed in those of Yang, et al., who observed that IL-6-induced STAT3 signaling in lung epithelial cell lines lead to increased RARα expression, which was abrogated when the STAT3 DNA-binding domain was substituted by the corresponding STAT1 domain [46]. The importance of our results with respect to prostate cancer is that this disease is often refractory to retinoid therapy, the molecular basis for which is not known at this time. Our results gives possible insight into the mechanism of retinoid insensitivity, and might also indicate that treatment of prostate cancer with STAT3 inhibitors and with retinoids may be beneficial.

In terms of androgen receptor function, S3c expression in BPH cells changed their response to androgens so that BPH-S3c cells were no longer stimulated by DHT, and the growth of BPH-S3c cells was not inhibited by flutamide treatment (Table 2). These findings with respect to the androgen receptor and responses to DHT and flutamide are especially important, as it may be the one of the first indications of a direct effect of STAT3 on androgen receptor responses, and may indicate a possible molecular mechanism for the development of the hormone-refractory state in prostate cancer patients. The progression to androgen-independence has been found to be associated with IL-6, with c-myc expression, and with insulin-like growth factors, all of which can signal through the activation of STAT3 [25-28]. It has been postulated that cross-talk between STAT3 and the androgen receptor plays a role in the development and maintenance of the hormone-refractory state in prostate cancer [47]; our data indicate that persistently-activated STAT3 may obviate the need for expression of the androgen receptor, since the androgen receptor did not respond to either DHT or F in S3c-transfected BPH-1 cells (Table 2). Further work is warranted in this area.

Prior to performing in vivo tumorigenicity experiments, we wanted to see if S3c-transfected cells could grow in soft agar as clones. We observed that S3c expression in NRP-152 cells allowed them to grow as clones in soft agar (Table 4). However, even though 152-S3c cells grew in soft agar, a phenotype usually consistent with tumorigenicity, in 3 out of 3 experiments we failed to observe tumors in more than 20% of the mice, and these tumors were not more than 1 mm in diameter (data not shown). Therefore, we concluded from these data that persistent expression of activated STAT3 alone was not sufficient to produce tumorigenicity in prostatic epithelial cells, although it had been sufficient in NIH-3T3 cells, as previously reported [40]. Furthermore, recent observations by Zhang and coworkers point to an important function for STAT3 in both tumorigenesis and metastasis formation in leiomyosarcoma [48], due to signaling by hepatocyte growth factor/scatter factor. Among the candidate genes regulated by STAT3 in this regard are matrix metalloproteinase-2, which is essential for tumor invasion and metastasis formation [49]. Perhaps STAT3 cooperates with another factor regulated by hepatocyte growth factor/scatter factor, which is not expressed by either NRP-152 or BPH-1 cells. Only more experiments will reveal whether this is the case. Indeed, we are planning experiments to see what genes are regulated by S3c, to gain insight into the phenotypic changes induced by S3c expression. For example, very recently it was reported that STAT3 and the microphthalmia-associated transcription factor were both required for optimal upregulation of *c-fos*, and subsequent tumorigenicity, in NIH-3T3 cells [50]. Whether the prostatic lines NRP-152 or BPH-1 express microphthalmia-associated transcription factor has not been determined; the levels of *c-fos *in S3c-transfected lines can be determined. As well, Dechow and coworkers reported that transfection of S3c into mammary epithelial cells rendered those cells tumorigenic in irradiated SCID mice [51]; whether our results are an indication of a difference between mammary epithelial cellls and prostatic epithelial cells or a reflection of irradiated vs. non-irradiated SCID mice remains to be elucidated. As more information is revealed about gene expression changes that accompany the progression of prostate cancer from the benign to the hormone-refractory state, the other genetic changes needed for tumorigenicity of S3c cells should be revealed.

## Conclusions

Our data indicate that transfection of NRP-152 and BPH-1 prostatic epithelial cells with a gene for persistently-activated STAT3, S3c, changed the phenotype of the cells into one resembling a malignant phenotype, thereby giving even more importance to the role of activated STAT3 in the transformation of normal cells into neoplastic cells. Importantly, we found that cells expressing S3c depended on its continued expression for survival. Two kinds of evidence are presented: first, S3c-transfected cells became sensitive to the effect of antisense STAT3 oligonucleotide. When transfected with antisense STAT3, both BPH-S3c and 152-S3c underwent apoptosis. Second, the S3c-transfected cells were not sensitive to the commonly-used STAT3 inhibitors, which are really JAK inhibitors, because activation of STAT3 by the upstream JAK is not required when S3c is expressed. We observed that growth factor-dependent NRP-152 cells grew without growth factor supplementation when transfected with S3c gene, whereas the medium for vector-transfected NRP-152 cells still required supplementation with growth factors. Moreover, we observed that 152-S3c cells grew in soft agar, whereas neither vector-transfected nor untransfected NRP-152 cells did. Furthermore, we observed that the expression of RAR subunits in 152-S3c cells was different from vector-transfected and untransfected NRP-152 cells, and that the changes were consistent with what we previously observed in specimens from prostate cancer patients, as well as in primary prostatic epithelial cells compared with prostate cancer cell lines [34,35]. These data may have implications for the relative lack of sensitivity of PCA to retinoid therapy. As for BPH-1 cells, which do not require growth factor supplementation, we observed that when transfected with S3c, this cell line lost its responses to testosterone and to the testosterone antagonist flutamide. Neither of these changes was observed in vector-transfected BPH-1 cells. However, neither S3c-transfected cell line was tumorigenic when injected into SCID mice, leading us to conclude that additional genetic changes are possibly needed for tumorigenicity in prostate cells.

## Methods

### Cell Lines

NRP-152 and NRP-154 cells were the gift of Dr. David Danielpour, Ireland Cancer Center, University Hospitals, Cleveland, OH [36]. Growth factor-dependent NRP-152 cells were grown in DMEM/Ham's F12 medium (1:1; GIBCO) supplemented with 10% newborn bovine serum (Hyclone), 2 mM glutamine (GIBCO), epidermal growth factor (20 ng/ml), insulin (5 μg/ml), dexamethasone (0.1 μM) and cholera toxin (10 μg/ml; all, Sigma), pH 7.3 (152 medium). NRP-154 cells were grown in DMEM/Ham's F12 medium plus 10% newborn calf serum (154 medium). Growth factor-independent BPH-1 cells [37,38] were the gift of Dr. Simon Hayward, Vanderbilt University, Nashville, TN. They were grown in RPMI-1640 medium supplemented with 10% newborn bovine serum. For transfections, cell were seeded into wells of 6-well plates and grown until 50–80% confluent monolayers of cells were present, as assessed by observation under inverted phase-contrast microscopy.

### Transfections

Derivation of the pBABE-S3c plasmid containing a constitutively-activated STAT3 gene, S3c (gift of Dr. Jacqueline Bromberg, Memorial Sloan-Kettering Cancer Institute) has been previously described [14,45]. The S3c gene was excised along with its FLAG tag, and inserted into pIRES-EGFP (Clontech), resulting in the plasmid called pIRES-S3c. For stable transfections, Clonfectin reagent (Clontech) was mixed with plasmid DNA (6 μl Clonfectin and between 1 and 3 μg plasmid), according to the manufacturer's instructions. The complete medium was removed from the plates of cells and replaced with 1.8 ml IMDM (Invitrogen). The Clonfectin-plasmid mixtures (100 μl) were added to the cells; replicate cultures of cells received Clonfectin only at the time of transfections. The plasmid-Clonfectin mixtures were left on the cells in the incubator for 4 hr, at which time the supernatant fluids were aspirated and replaced with 5 ml/well pre-warmed complete medium. Twenty-four hr following transfections, G418 (Invitrogen) was added at a final concentration of 800 μg/ml. The medium plus G418 was replaced 3 times/wk until no surviving cells were observed on the Clonfectin-only wells, usually 2 weeks. At that time, G418 was added at 100 μg/ml to maintain the transfected cells. When the transfected cells reached confluence, they were used for further analyses. Table 1 gives a summary of transfected cells and phenotypes obtained.

For transient transfections, LipoFectamine 2000 in Opti-MEM I medium (both, Invitrogen) was used according to the manufacturer's directions. For subconfluent (~50%) cells, 2 μl of LipoFectamine 2000 was used with varying amounts of antisense or sense STAT3 oligonucleotide (gift of Dr. James Karras, ISIS Pharmaceuticals). The oligonucleotides were left on the cells for 6 hours before cell culture medium supplemented with 30% was added to each well. Cells were incubated until assays were performed.

### Limit-Dilution Cloning

In order to analyze clonal populations of cells, transfected cells (pIRES-S3c or pIRES-EGFP) were harvested, diluted to 10 cells/ml in complete medium, and seeded into microtiter plates at 100 μl/well. The total volume of each well was brought to 200 μl with additional medium, and the plates were incubated until growth of seeded cells was observed, usually at 10 days to 2 weeks.

### Determination of Stable Transfection by Expression of FLAG in 152-S3c and BPH-S3c Cells by Intracellular Flow Cytometry

Expression of the FLAG epitope engineered onto the constitutively-activated STAT3 gene in transfected NRP-152 cells was performed by intracellular flow cytometry, as described [52]. Briefly, 152-S3c or BPH-S3c cells were harvested, washed, and fixed in 4% paraformaldehyde/PBS (Pharmingen) for 30 min on ice. Fixed cells were washed and permeabilized with 0.1% sapononin (Pharmingen) for 15 min at room temperature, then washed. Mouse monoclonal Ab M1 to FLAG (Sigma) was added (1 μg/10^6 ^cells/100 μl permeabilization buffer) to the cells for 1 hr on ice. The cells were washed 3 times, then incubated with phycoerythrin (PE)-labeled goat anti-mouse F(ab_2_)' (Caltag) for 1 hr on ice in permeabilization buffer. After washing 3 times, cells were resuspended in 1 ml PBS and analyzed on a Becton-Dickinson FACScan. CellQuest software was used to acquire and analyze the fluorescence. The Kolmogorov-Smirnov 2-sample test was used to determine the level of significance of the change in fluorescence intensity between control-stained (F(ab_2_)'-stained only) and Ab-stained populations of cells, thereby ascertaining that the populations observed in the histograms were truly separate populations of cells [53].

### Immunoprecipitation/Western Blot Studies

For immunoprecipitation, cells lysed in Lysis Buffer (10 mM PBS, pH 7.4, 1% NP-40, 0.5% sodium deoxycholate, 0.1% sodium dodecylsulfate (SDS), 1 mM sodium orthovanadate, 1 mM phenylmethyl-sulfonyl fluoride, 40 μg/ml aprotinin) were precleared with Protein A/G agarose (Santa Cruz Biotechnology), then precipitated with 1–5 μg rabbit Ab (Cell Signaling or Pharmingen) plus Protein A/G (Santa Cruz Biotechnology) agarose overnight. After washing, the beads were eluted by heating in Laemmli buffer for 5 min at 95°C, followed by electrophoretic separation on 12% SDS-polyacrylamide gels (Novex Nu-PAGE pre-cast gels). Transfer of separated protein species to nylon membrane (Millipore) was followed by blocking in 10% non-fat dry milk in TBST (50 mM Tris HCl, pH 7.4, 150 mM NaCl, 0.3% Tween 20). Incubation of the membrane with rabbit Ab was followed by incubation with alkaline phosphatase-linked goat anti-rabbit antibody (Amersham ECF kit). After addition of substrate from the kit, the membranes were read by the Typhoon imager, with ImageQuant software for resolution of images (Molecular Dynamics).

### Measurement of In Vitro Growth of Cells

NRP-152, NRP-154, BPH-1, and transfected cells were seeded at 10^3^cells/well in microtiter plates in appropriate medium, as indicated. After 48 hr, 15 μl MTT (Sigma; 25 μg/ml) was added to each well for 4 hr, then the resulting formazan was dissolved in 0.1% SDS. Absorbance was determined at 570 nm on a Dynatech microplate reader. Statistical determinations of significance were performed by unpaired Student t-test for multiple independent assays, using GraphPad software.

### Determinations of Androgen Insensitivity and Presence of Retinoid Receptors

The effect of dihydrotestosterone (DHT) as growth agonist, and the effect of flutamide (F) as growth antagonist, was assessed by use of the MTT assay described above. DHT and F were obtained from Boeringer-Mannheim, and cells were treated with 1 or both drugs at concentrations ranging from 1 to 100 nM for DHT, and 0.1 to 3 μM for F. These are within the published ranges of efficacy for these drugs [34,54]. Vehicle controls were included. Replicate plates were harvested at 24, 48, 72, and 96 hrs after treatment.

Northern blot hybridizations to detect the retinoid receptors RARα, RARβ, and RARγ were performed as previously published [34]. In brief, RNA was isolated from cells using RNAEasy (Qiagen) and quantified spectrophotometrically. RNA was separated by size on agarose gels, then transferred to nitrocellulose membranes (Schleucher & Schuell). The probe was labeled with ^32^P-dCTP (New England Nuclear), then allowed to hybridize to the blot overnight in hybridization buffer. After washing, hybridization was detected by use of a PhosphoImager (Molecular Dynamics).

### Apoptosis Assays

Forty-eight hr after transient transfection, cells were harvested using Enzyme-Free Cell Dissociation Buffer. After two washes with PBS, they were stained with FITC-annexin V (5 μl/10^6 ^cells; Caltag) for 15 min at room temperature. Apoptotic cells (cells staining with FITC-annexin V) were quantified by measuring green fluorescence in FL1 on the flow cytometer. In some experiments, cells were also stained with propidium iodide (PI), which is detected by the FL3 detector. CellQuest software was used to acquire and analyze the data on a Becton-Dickinson FACScan flow cytometer. For studies using the tyrphostin JAK2 inhibitor AG490 (Calbiochem), the dissolved compound was added to subconfluent cells, as described [11]. A vehicle control was included for the 0 μM concentration. Forty-eight hrs later, cells were harvested and processed for quantification of apoptosis by annexin V binding and PI incorporation.

### Assay for Growth in Soft Agar

Transfected cells were subjected for growth in soft agar to assess their change in phenotype with regards to colony formation. After selection and cloning, 10^4 ^cells were trypsinized and washed in Ca2+/Mg2+-free PBS (Life Technologies) and plated in 1 ml of medium plus serum without supplements containing 0.3% (w/v) Noble Agar (Difco/Becton-Dickinson) over a 2 ml layer of the same medium with 0.6% agar in six-well plates. The number of colonies was counted using low magnification microscope (4×) after 10 days.

### In Vivo Tumorigenicity Studies

Our protocol was reviewed and approved by the Institutional Animal Care and Use Committee of UMDNJ. Severe-combined immunodeficient (SCID) mice (Charles River Laboratories) were obtained at 5 weeks of age, and acclimatized in the barrier vivarium for 1 week. At that time they were injected subcutaneously with 8 × 10^6 ^S3c or vector-transfected (pIRES-EGFP) control cells. Each group consisted of 5 animals. In some experiments, the cells were mixed with Matrigel (Collaborative Research) prior to injection. Tumor growth was monitored weekly using engineer's caliper's to measure the 2 perpendicular diameters, over the course of 12 weeks.

## List of abbreviations

STAT signal transducer and activator of transcription

cSTAT3 constitutively-activated STAT3

JAK Janus activated kinase

PCA prostate cancer

S3c constitutively-activated STAT3 gene having a Cys substitution

FLAG an immunogenic peptide fused to gene of protein to be expressed for identification and/or purification purposes

SCID severe combined immunodeficient

PBS phosphate-buffered saline

DMEM Dulbecco's modifcation of Eagle's medium

IMDM Iscove's modification of Eagle's medium

FITC fluorescein isothiocyanate

PE phycoerythrin

PI propidium iodide

FL1, 2, or 3 fluorescence detectors on a flow cytometer that collect fluorescence data within a

set range of wavelengths, FL1 being the lowest and FL3 being the highest

EGFP enhanced green fluorescence protein

RAR retinoic acid receptor

DHT dihydrotestosterone

F flutamide

## Authors' contributions

HFH conceived of the retinoic acid receptor subunits experiments, and perofrmed the northern blot hybridizations. TFM performed the growth factor dependence and growth rate experments, performed some of the in vivo experiments, and prepared cells for flow cytometry. PS performed some of the transfections, participated in the in vivo experiments, the western blots, and prepared cells for flow cytometry. ABB made the pIRES-S3c plasmid from pBABE-S3c, and performed the soft-agar cloning experiments. BEB conceived of the project, performed most of the transfections, performed some of the in vivo experiments, and performed all flow cytometry acquisitions and analyses. All authors read and approved the manuscript.
